# From data silos to insights: the PRINCE multi-agent knowledge engine for preclinical drug development

**DOI:** 10.3389/frai.2025.1636809

**Published:** 2025-08-19

**Authors:** Carlos Henrique Vieira-Vieira, Sarang Sanjay Kulkarni, Adam Zalewski, Jobst Löffler, Jonas Münch, Annika Kreuchwig

**Affiliations:** ^1^Bayer Research and Development, Pharmaceuticals, Preclinical Development, Berlin, Germany; ^2^Thoughtworks Technologies (India) Private Ltd., Pune, India; ^3^Bayer Pharma Drug Innovation, Technology and Engineering, Leverkusen, Germany; ^4^Bayer Digital Transformation and IT Pharma, Berlin, Germany

**Keywords:** pharmaceutical industry, preclinical, large-language model, chatbot, retrieval-augmented generation, generative artificial intelligence, regulatory document generation, agentic artificial intelligence

## Abstract

The pharmaceutical industry faces pressure to improve the drug development process while reducing costs in an evolving regulatory landscape. This paper presents the Preclinical Information Center (PRINCE), a cloud-hosted data integration platform developed by Bayer AG in collaboration with Thoughtworks. PRINCE integrates decades of structured and unstructured safety study reports, leveraging a multi-agent architecture based on Large Language Models (LLMs) and advanced data retrieval methodologies, such as Retrieval-Augmented Generation and Text-to-SQL. In this paper, we describe the three-step evolution of PRINCE from a data search tool based on keyword matching to a resourceful research assistant capable of answering complex questions and drafting regulatory-critical documents. We highlight the iterative development process, guided by user feedback, that ensures alignment with evolving research needs and maximizes utility. Finally, we discuss the importance of building trust-based solutions and how transparency and explainability have been integrated into PRINCE. In particular, the integration of a human-in-the-loop approach enhances the accuracy and retains human accountability. We believe that the development and deployment of the PRINCE chatbot demonstrate the transformative potential of AI in the pharmaceutical industry, significantly improving data accessibility and research efficiency, while prioritizing data governance and compliance.

## Introduction

The pharmaceutical industry faces pressure to accelerate drug development timelines and reduce costs, while ensuring the delivery of safe, effective therapies. Preclinical development teams play a crucial role in this process by identifying safety risks and optimizing drug candidates before human trials, thereby minimizing late-stage attrition. However, data-driven decisions are often hindered by fragmented data scattered across multiple systems with varying annotation quality. This often limits these team’s ability to conduct comprehensive safety analysis efficiently, requiring significant manual effort (Carfagna et al., 2021).

Recent advancements in Large Language Models (LLMs) have revolutionized the landscape of data analysis in various fields, including pharmaceuticals (Tian et al., 2024; Chakraborty et al., 2025). These models have demonstrated remarkable capabilities in understanding and generating natural language and facilitating complex data interpretation based on input prompts (Sivarajkumar et al., 2024). More recently, the development of agentic workflows has enhanced the ability of LLM-based systems (Xi et al., 2023; Schneider, 2025). Beyond answering questions, multi-agent systems - wherein several LLMs interact with each other independently from human control - are vastly increasing research capabilities (Guo et al., 2024; Su et al., 2025).

The pharmaceutical industry has recognized the need for integrated data platforms to overcome these challenges. As highlighted by (Steger-Hartmann et al., 2023), platforms like PRINCE (Preclinical Information Center) at Bayer and SDI (Safety Data Integration) at Roche were developed as central hubs for accessing and analyzing preclinical safety data. In this manuscript we present how Bayer’s PRINCE platform has evolved into a custom-designed chatbot through the implementation of an LLM-based multi-agent system. With real-world use cases, we demonstrate how the PRINCE chatbot enables comprehensive data retrieval and interpretation, enhancing the decision-making process. In addition, we highlight how its collaborative agent framework executes complex tasks, including data interpretation and drafting of documents. Collection of feedback from end-users and its implementation in guiding platform development is reported. The findings presented herein highlight the chatbot’s ability to leverage internal data to write regulatory-critical documents, thereby supporting Bayer’s commitment to increase productivity while adhering to the industry’s best practices.

## Methods

### Data aggregation, integration and governance

The PRINCE platform was developed to enable data aggregation, integration, and governance. Data and metadata from Laboratory Information Management Systems (LIMS) and other internal research applications were collected and stored in AWS S3 (Amazon Web Services Simple Storage Service), formatted similarly to the CDISC SEND (Standard for Exchange of Nonclinical Data) data standard for preclinical studies (SEND | CDISC, 2021). An access control system was put in place to ensure compliance with internal data classifications. Study metadata was aggregated and harmonized semi-automatically by data scientists and toxicology experts, utilizing CDISC SEND controlled terminology. Mapping lists were generated and integrated into data pipelines for extraction, transformation, and loading.

Study reports, including text, tables, and figures were processed using optical character recognition for scanned and native PDFs documents. Extracted content was stored as JSON files, preserving the original document structure. Content was split into chunks using a recursive character text splitter strategy, with parameter tuning for optimal retrieval. Metadata, such as accession identification numbers and study-specific information, were added to enhance context. Chunks and metadata were embedded using openAI’s *text-embedding-3-large* into Amazon OpenSearch Service.

### PRINCE chatbot multi-agent architecture

**Figure 1:**
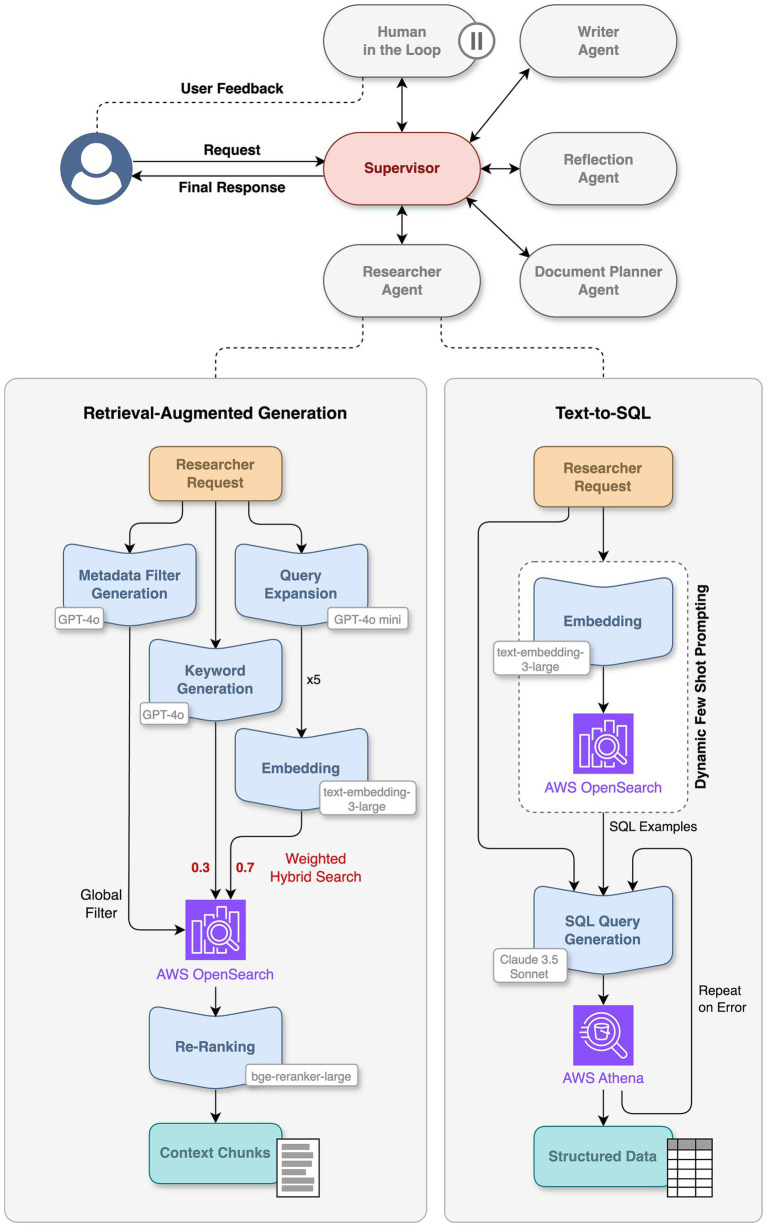
PRINCE multi-agent architecture. Upon receiving a user request, the supervisor agent coordinates specialized agents to generate the response. These agents utilize tools such as retrieval-augmented generation for extracting relevant context chunks from unstructured data and Text-to-SQL for retrieving structured data from AWS Athena. A human-in-the-loop node is used to request user feedback when necessary. LLMs used are indicated next to the respective steps in the workflow. Parallel executions for query expansion are not shown for clarity but indicated with “5x.”

The chatbot was deployed within PRINCE utilizing a multi-agent system managed with LangGraph (2025). A Supervisor Agent orchestrates the more specialized agents for complex user requests (Figure 1). Upon submission, the Supervisor Agent initiates an LLM call to analyze user intent and identify tools and data required to answer the request. If data is required, information is retrieved using Retrieval-Augmented Generation (RAG) and Text-to-SQL (see Information Retrieval Techniques section). The Reflection Agent evaluates the sufficiency of collected data through iterative LLM calls, identifying gaps and generating follow-up requirements for the Supervisor Agent. This process is repeated until sufficient data is obtained as judged by the Reflection Agent, or a fixed number of repetitions were retried. The Supervisor Agent then generates the final answer, using the extracted data and relevant document chunks as context within a dedicated LLM prompt. For transparency, citations linking back to original documents or relevant metadata to support the final answer are included in the response.

If a user requests drafting of a document such as the Investigational New Drug (IND) report, a Document Planner Agent is invoked by the Supervisor Agent before generating the final response. This specialized agent is designed with an understanding of the structure and content of various IND report sections (US Food and Drug Administration, 2020), using a library of guidelines embedded as LLM prompts in a vector database. The Document Planner Agent compares the user request against this database to retrieve relevant prompts from an in-house curated library, enhancing them with information from the Supervisor Agent. A human-in-the-loop (HITL) approach (Kumar et al., 2024) then enables users to review and edit prompts before they are used by the Supervisor Agent, ensuring the final response is accurate and relevant.

For calls to LLMs, we programmatically interface Bayer’s internal GenAI platform *myGenAssist*, as previously described elsewhere (Benaïche et al., 2025), and also utilize the *Linguist* platform, Bayer’s Natural Language Processing toolbox, enabling access to the latest models in a secure and efficient way. OpenAI’s *GPT-4o* model is used unless specifically noted with temperature set to 0. This choice leverages a dedicated subscription model offering optimized cost efficiency and high performance.

### Information retrieval techniques

#### Text-to-SQL

For structured data retrieval from AWS Athena a Text-to-SQL translation method was developed in-house (Figure 1). To convert natural language requests into SQL queries, an LLM (*Claude 3.5 Sonnet*) analyzes user requests to identify data points and filters. The LLM prompt includes the description of relevant database schema components, dynamically reduced for large schemas. Instructions given include essential columns, like study identifiers, and permit only SELECT operations. Accuracy of SQL generation is enhanced through dynamic few-shot prompting (Dynamic Few Shot Prompting in LangChain, 2025) using curated examples of natural language queries and their SQL translations from an in-house curated library in AWS OpenSearch, continuously extended and updated with user feedback. The generated SQL query is executed against the PRINCE database in Amazon Athena. If execution fails, the query is recreated up to three times with the failed SQL query, the error messages and the original request as context for the next prompt. Successful queries, including a limit of 50 records, are passed on as a dictionary to the Supervisor Agent to evaluate if further processing, e.g., additional data or human feedback, is needed.

#### Retrieval-augmented generation

For retrieving unstructured data from study reports, PRINCE employs a RAG (Lewis et al., 2020) pipeline targeting its vector database (Amazon OpenSearch Service), utilizing a weighted hybrid search approach that combines semantic vector similarity and keyword-based matching (Figure 1). Initially, the user’s natural language query is processed by a *GPT-4o* model prompted to extract both metadata filters and keywords for search. Metadata filters are applied directly to the vector database to refine the search space. Subsequently, to enhance retrieval comprehensiveness, a *GPT-4o mini* model generates five semantically similar queries based on the original question, triggering parallel hybrid searches against the filtered vector database.

Within each individual hybrid search, results from semantic vector similarity using a kNN algorithm are adjusted with a weight of 0.7, while keyword search results receive a weight of 0.3. These weights were determined through evaluations aimed at optimizing scores for a specific dataset. The chunks retrieved from all parallel searches are aggregated and unique chunks are ranked based on their weighted scores, resulting in an initial set of approximately 20 chunks. This set is then reranked by a *bge-reranker-large* cross-encoder model (Xiao et al., 2023), evaluating relevance against the original question to select the most relevant chunks. These selected chunks, along with the original user query, form a contextualized prompt for a *GPT-4o* model instructed to synthesize this information into a coherent answer, complete with citations to the source documents.

### Evaluation

To monitor performance and reliability, datasets of curated questions and reference answers developed by domain experts in-house were evaluated frequently. Each evaluation dataset covered different types of questions ranging from lower to higher complexity and were managed using Langfuse (2025). A custom evaluation script processed each dataset question through the PRINCE chatbot, comparing generated responses to reference answers. Multiple quantitative metrics were calculated to assess chatbot performance, including faithfulness, answer relevancy, context precision, factual correctness, and semantic similarity (Es et al., 2024). Evaluations were also conducted at intermediate stages in the multi-step process of the agentic system, complementing the end-to-end performance assessments. To rapidly identify and resolve issues in the production environment, including hallucinations, evaluations were conducted daily on user requests and chatbot responses. Due to the absence of pre-defined reference answers in these evaluations, only faithfulness, context precision and answer relevancy were calculated.

### Error handling and recovery

Given the complexity inherent in the multi-step agentic workflow, robust error handling and recovery mechanisms were engineered to ensure system reliability, provide a seamless user experience, and enable graceful recovery from failures without requiring complete workflow restarts. State persistence is a cornerstone of this approach: the ‘Agent State’, representing agent progress through the workflow, was stored in a Postgres database, while other application state components like logs, intermediate steps, and citations were stored in DynamoDB.

The system incorporates built-in retries, configured to automatically re-execute a transiently failed step a predefined number of times. Additionally, users can manually initiate a retry for a failed request via the interface; the system then leverages the persisted state to continue from the precise point of failure, skipping previously successful steps. To further bolster reliability against external model issues, custom LLM fallback handling is implemented, automatically switching to an alternative LLM from a different inhouse provider (using the same model but from a different source) if a primary LLM call fails after a few retries. The LangGraph (2025) framework was used for state management and error handling.

## Results

The data integration platform PRINCE has been under development since 2020. With the goal of making the right data available at the right time, our approach is to become the one-stop shop for preclinical data at Bayer. Since the release of a minimal viable product in 2021, many features have been developed, including the addition of the chatbot in March 2024 and the implementation of a multi-agent system in November 2024. In this section, we describe in detail the developmental process of PRINCE, highlighting use cases from Bayer’s preclinical department.

### Search: breaking the data accessibility barrier

PRINCE integrates a vast repository of over 18,000 in-house studies ingested from the many in-house data source systems. The platform is designed to combine structured and unstructured data from toxicology, safety pharmacology and drug metabolism and pharmacokinetics studies and its endpoints (such as 4-week systemic toxicity, toxicokinetic or cardiovascular studies). Because we initially focused enabling our users to search for data, we called it the “Search” stage.

Data in PRINCE can be accessed programmatically or through an intuitive web-based user interface by setting specific search filters, promptly searching for relevant data from studies recorded in any of the data sources linked to PRINCE. For data analysis, pre-set visualizations are provided aside data exporting functionalities. Filtering parameters have been added on-demand to the user interface based on user feedback and currently include:

- Filters such as study and compound identifiers used internally and externally (e.g.: CAS numbers), research area, and the molecular target of the test compounds
- Filters related to the study design, such as route of administration, drug vehicle, animal species, and dosing duration
- Filters related to study findings, such as organs/tissues analyzed, type of finding observed, and laboratory tests performed
- Free-text keyword matching within title, summary and conclusion of the study report (e.g., “liver inflammation AND liver weight” in summary and conclusion would retrieve studies which have both terms in the selected context)

### Ask: leveraging AI for insightful analysis

While PRINCE users can access unstructured data from preclinical study reports at the Search stage, navigating through text, tables and figures can be challenging and result in suboptimal use of this data. Therefore, as the next stage in the PRINCE development, we implemented an LLM-powered chatbot in which users’ natural language requests are answered based on both structured and unstructured data available within PRINCE.

Building on the approach implemented in the user interface for searching structured data, the PRINCE chatbot analyzes the natural language user request to first identify filtering criteria. For example, a search for “studies performed in rats,” is translated into parameter filter “species” equals to “rat.” Structured data retrieved from PRINCE with these filters is then passed to the chatbot.

For the chatbot to access unstructured content from preclinical study reports and other documents within PRINCE, the content of these documents was split into partially overlapping chunks and embedded for storage into a vector database (see Methods section). Due to the large amount of unstructured data available in PRINCE, the chatbot first narrows down the search space by applying the filters identified from the user request to the chunks database. Next, most relevant chunks are identified by the similarity between chunks and the user request. Importantly, this also enables precise determination of the origin of specific information, providing accurate links to the original document paragraph, and ensuring transparency by enabling users to verify the information in the original study results.

**Figure 2:**
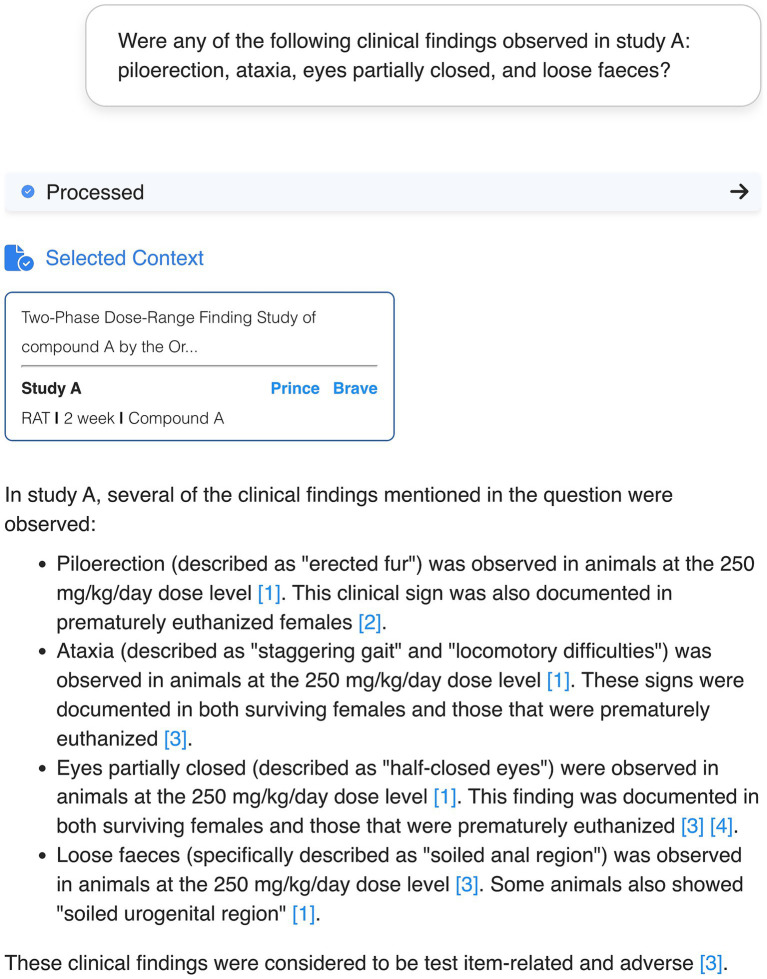
User interface of an exemplary conversation with the PRINCE chatbot. The user request includes specific clinical symptoms (“piloerection,” “ataxia,” “eyes partially closed” and “loose faeces”). In its reply, the chatbot identified the requested terms based on semantically similar terminology (“fur erected,” “staggering gait,” “half-closed eyes” and “soiled anal/urogenital region”). The numbers in brackets indicate the specific chunks used as source for the information provided by the chatbot. The context selected by the chatbot to build the answer is displayed in the blue box referring to the original sources. Confidential information such as study IDs or compound names was obscured.

This development stage was called the “Ask” stage because researchers can post requests in natural language that are interpreted semantically by the chatbot. For example, when specific clinical observations such as “piloerection” or “ataxia” in a specific study are requested, the chatbot interprets these keywords by identifying synonyms and clinically similar observations, such as “Fur erected” and “Staggering gait,” respectively (Figure 2). This indicates that the chatbot performs semantic analysis of the request, going beyond the simple keyword matching for data selection.

### Do: transforming insights into actionable strategies

The latest phase of PRINCE’s chatbot evolution focused on enabling it to take sophisticated actions based on the user request and was, therefore, named the “Do” phase. For that, we implemented a multi-agent workflow that supports answering complex questions, drafting regulatory documents, designing new studies and planning experiments (Figure 1). In the “Do” phase, the multi-agent system goes beyond consolidating parallel actions into a single process that is reliable and reproducible. It is, in fact, a generalized approach to orchestrate complex processes involving multiple steps that are interdependent. To exemplify these capabilities, we present results on a complex user request to write the draft of an IND document.

**Figure 3:**
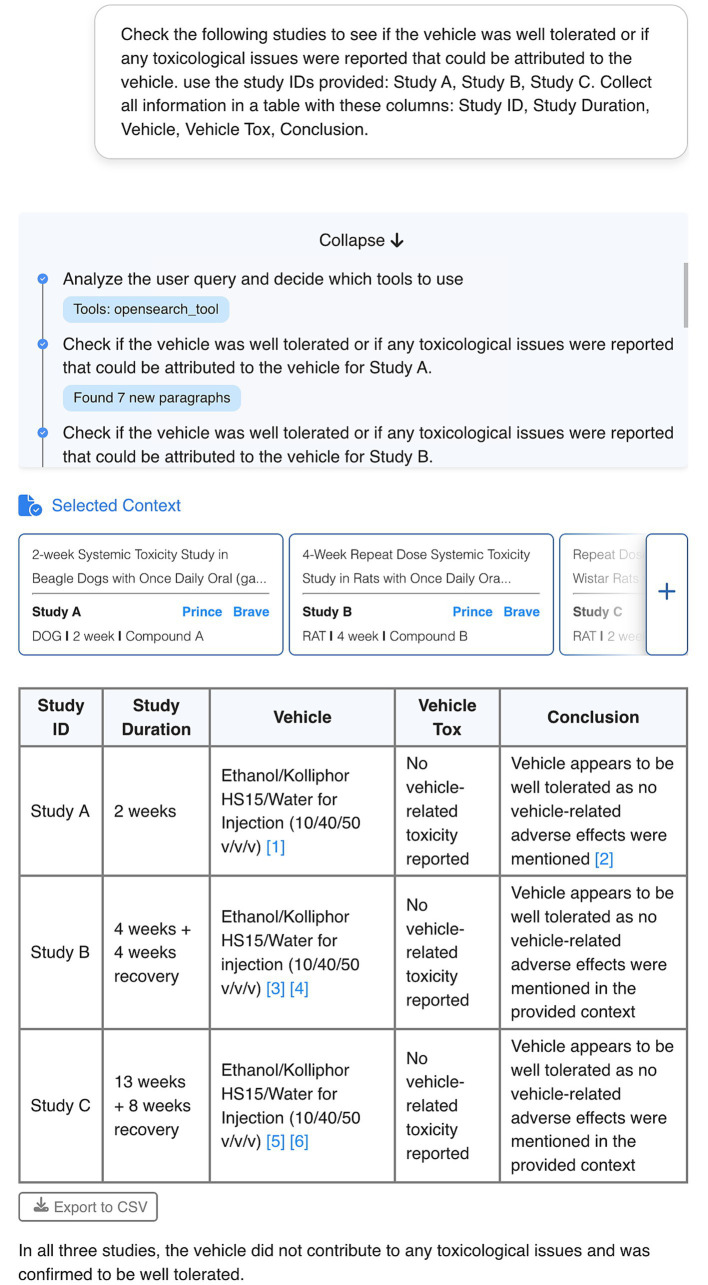
User interface of an exemplary conversation with the PRINCE chatbot. In this complex request, the user asks the chatbot to interpret the vehicle tolerability in a set of studies. The chatbot breaks down the task for each study in the request, identifying 4–8 relevant chunks of content per study. The chatbot follows the tabular format requested by the user when producing the final answer. The numbers in brackets indicate the specific chunks used as source for the information provided by the chatbot. The context selected by the chatbot to build the answer is displayed in the blue boxes referring to the original sources. Confidential information such as study IDs or compound names was obscured.

As a first use case, we hypothesized that a chatbot with access to data would be able to assist users in interpreting it. When presented with a complex request to analyze the vehicle toxicity from data of several studies, instead of attempting to answer directly, the chatbot multi-agent system analyzed the request, recognizing that a complete answer required multiple steps (Figure 3). First, it broke down the request to retrieve the relevant information from each individual study and then processed each sub-request separately, finding approximately 4 to 8 relevant chunks of content for each study. This information was then collected and interpreted to generate a comprehensive response that was formatted in the requested tabular format. The ability to independently discern complex from simple requests and to execute the requested actions stepwise allows the chatbot to perform analytical tasks that would normally require significant manual effort from researchers.

Among the other agents in our chatbot, a Document Planner agent for drafting regulatory documents, such as IND or study reports, was implemented. Upon a request to draft an IND report, the chatbot writes the entire document one section at a time. It first retrieves relevant information from the user request that is handed over to the Document Planner agent to construct section-specific prompts based on an in-house curated prompt library. Before drafting any documents, the prompt is given back to the user for revision. This document drafting capability with human collaboration saves time, reduces editing mistakes and improves document adherence to regulatory standards, while also maintaining human accountability over document creation.

### Iterative development and user-centric feedback

The PRINCE platform has been developed using an agile methodology that emphasizes continuous user interaction and feedback. Features were introduced to users in dedicated showcase sessions involving short demonstrations. Open-door sessions were also offered, providing opportunities for users to interact within the user community and explore new functionalities together, while also bringing together users and the product development team. This approach also ensured that the platform was developed according to the business needs of our end-users.

To assess the impact of each developmental stage, we collected feedback from 15 to 20 frequent users across various roles within Bayer’s preclinical development department, employing mixed methods including structured interviews, usability testing sessions and training. General ease of use for both the PRINCE platform and its chatbot interface was graded 4 out of 5 (average ease of use score). Users valued the consolidated interface for accessing complex data, highlighting time savings and improved efficiency in information retrieval. Specifically, 75% of users reported a significant reduction in time spent searching for information. Quantitative data analysis showed a 30% average improvement in response times for complex queries upon implementation of the multi-agent system. However, the overall capability of the chatbot to fully meet user needs received a lower average score (3.1/5.0), highlighting the need for further improvements.

Qualitative feedback suggested that transparency of results is a main area for improvement. One main recurrent feature request was the need for in-text citations with reference to the paragraph and source document used for a particular claim in the response. User preferences for citation format leaned toward a Wikipedia-style format with clear reference numbering and readily accessible source links. This feature was readily implemented and is currently featured in the chatbot (Figures 2, 3).

## Discussion

The PRINCE platform represents a significant innovation in preclinical development, addressing the challenges of data management, analysis and reporting by consolidating structured and unstructured data from over 18,000 study reports. Here, we reported the evolution of the platform from a search tool to a comprehensive AI-driven research assistant empowering users to ask complex questions and take actionable steps, such as drafting compliance-critical documents.

Initially, the PRINCE platform focused on providing a centralized, searchable repository of preclinical data, consolidating information scattered across internal systems. However, relying solely on keyword searches proved insufficient and prone to overlooking critical data. The introduction of an LLM-based chatbot marked a significant step forward, as the chatbot accesses and interprets extensive amounts of structured and unstructured data. Most recently, the incorporation of a multi-agent system capable of supporting complex tasks greatly expanded the platform capabilities, while retaining human accountability.

The progression from “Search” through “Ask” to “Do” described here was driven by the increased need for efficiency and accuracy in data analysis and a commitment to building user trust through transparency and explainability. It also reflects a strategic shift from simply providing data access to actively supporting researchers in their decision-making and knowledge creation processes. Also, as highlighted by the presented use cases and the user feedback collected throughout the development process, PRINCE saves time and resources of users, streamlining preclinical drug development, and contributing to mitigate the risk of late-stage failures.

### Building trust through transparency and explainability

The development of the PRINCE chatbot focused on addressing user concerns regarding the trustworthiness and potential for hallucinations in AI chatbots (Li et al., 2024). This user-driven, trust-focused approach has led to a strong emphasis on transparency. One example of how transparency is achieved is the log-tracking feature, where each step executed by the chatbot is displayed to the user. Intermediate steps, including formulated sub-requests, tools utilized (e.g., RAG, Text-to-SQL) and document selection are also displayed. This “show your work” approach provides visibility into the chatbot’s reasoning process, allowing users to follow the steps taken to arrive at the final answer.

Transparency was also greatly improved by providing precise, in-text citations referencing the original study report fragments and structured metadata used by the chatbot. That way, within the chatbot’s interface, users can hover over sentences in the response to view the corresponding citation, including a link to the specific source document, its page number, and the exact quote extracted. This granular level of traceability is crucial for verifying the chatbot’s claims. Regarding PRINCE’s multi-agent architecture, the interplay between agents is also visible to the user in the logs, exposing how complex queries are decomposed, processed, and synthesized into coherent responses. The Reflection Agent’s role in evaluating information sufficiency and generating follow-up questions is of particular interest to users, clarifying the system’s iterative refinement strategy.

### Expanding PRINCE with external and internal data and self-correction

A recent user survey indicates that, although a powerful tool in assisting Bayer experts, improvements to the chatbot could further support end-users. Therefore, we plan on expanding PRINCE’s knowledge base with both internal (e.g., by ingesting decades-worth of archival documents not available in current document management systems) and external sources, such as scientific literature. Crucially, PRINCE’s transparency features would extend to external sources, ensuring all claims remain explainable and traceable. Incorporating in-house early research and clinical data is particularly relevant, enabling valuable translational analyses. Furthermore, similar document drafting use cases across other stages of drug development may also be tackled withing PRINCE.

Importantly, the accuracy and completeness of structured data in PRINCE are crucial for correct retrieval of data. Existing metadata from multiple data sources was often found to be incomplete and sometimes incorrect due to various operational reasons. To address this issue, we are currently investigating methods to extract accurate metadata, such as study identifiers, compound names, species, routes of administration, dosage information, and clinical findings, directly from preclinical study reports. This improvement of the underlying metadata by the multi-agent system may significantly improve its own ability to answer user requests.

### PRINCE in the context of evolving regulatory guidelines

While regulatory bodies, including the FDA (US Food & Drug Administration), are beginning to acknowledge the potential value of AI-assisted document generation (US Food and Drug Administration, 2025a), clear guidelines are still evolving. In fact, the FDA recently completed its first AI-assisted scientific review pilot and announced a fast agency-wide AI rollout timeline, with FDA scientists reporting that generative AI tools have reduced review tasks from days to minutes (US Food and Drug Administration, 2025b). It is also clear that integration of AI technologies like PRINCE into regulatory document preparation represents a significant opportunity, but human accountability must remain key. In this context, our transparent, citation-based approach serves as a powerful tool for gathering and organizing information, while ensuring that human experts maintain oversight and accountability. Also, by implementing a human-in-the-loop component within the Document Planner agent, control over the system is shared with the end-user. Therefore, scientists using PRINCE for regulatory document preparation benefit from its ability to efficiently compile relevant data and draft structured content, but retain responsibility for reviewing, validating, and finalizing all submissions.

Recently, the FDA announced plans to phase out animal testing requirements for monoclonal antibodies and other biologic products (Poh and Stanslas, 2024; US Food and Drug Administration, 2025c). The FDA’s initiative aligns with broader industry trends toward alternative testing methods, as reflected in the FDA Modernization Act 2.0, which removes the mandatory requirement for animal testing in drug development (Han, 2023). This shift makes historical preclinical data even more valuable, as researchers extract maximum insights from existing studies while designing more targeted and efficient new experiments. However, the value of this data can only be realized if it is accessible, searchable, and analyzable, challenges that are directly tackled by PRINCE. Therefore, we envision that PRINCE may play a crucial role in Bayer’s development of alternative testing methods required to replace animal testing.

## Data Availability

The data analyzed in this study is subject to the following licenses/restrictions: internal data, not shareable. Requests to access these datasets should be directed to corresponding author.
